# Anemia as a mediator: bridging the frailty index and hip fractures in older Chinese populations

**DOI:** 10.3389/fpubh.2025.1558074

**Published:** 2025-04-23

**Authors:** Changqing Li, Xiaojiang Zhao, Lei Zhang, Chao Ma, Wenbo Zhang, Hong Ding

**Affiliations:** Department of Physical Education and Arts, Bengbu Medical University, Bengbu, China

**Keywords:** frailty index, anemia, hip fracture, CHARLS, older adults

## Abstract

**Background:**

Hip fracture is a significant global public health issue. The link and mechanisms between frailty index (FI) and hip fracture remain unclear. This research examined how anemia mediates the link between FI and hip fracture.

**Methods:**

The study analyzed data from the 2015 China Health and Retirement Longitudinal Study (CHARLS), which included 6,326 participants aged 60 and above. The mediating role of anemia in the relationship between FI and hip fracture was examined using bootstrap analysis and linear regression models.

**Results:**

After controlling for confounding variables, FI was positively associated with hip fracture (OR = 1.13, 95% CI: 1.09–1.16; *p* < 0.001). Anemia was also positively associated with hip fracture (OR = 1.88, 95% CI: 1.33–2.64; *p* < 0.001). Mediation analysis showed that anemia indirectly affected the relationship between FI and hip fracture, accounting for 18.95% of the total effect. Subgroup analysis showed that compared with non-frail and non-anemic participants, frail and anemic participants had a significantly increased risk of hip fracture (OR = 4.61, 95% CI: 2.80–7.61). However, no interaction between frailty and anemia was observed for hip fracture risk.

**Conclusions:**

The findings suggest that FI and anemia were positively associated with hip fracture, and anemia played a mediating role in the association between FI and hip fracture. Intervention based on exercise, nutrition and medical management can combat anemia and reduce FI and may be an effective way to prevent or delay hip fractures.

## 1 Introduction

Hip fractures are a major public health issue, predominantly affecting older people. These fractures are frequently referred to as the “final fracture of life” in this demographic, owing to their poor prognosis, numerous complications, and elevated mortality rates (1–3). Key risk factors for hip fractures in the older people include advanced age, gender, smoking, alcohol use, hypertension, diabetes, osteoporosis, poor vision, gait and balance problems (2–5). It frequently results in premature mortality, significant disability, limited ability to perform activities of daily living (ADL), and a decreased quality of life (6). Hip fractures are rare among younger individuals, as their occurrence is significantly correlated with advancing age, particularly in the older population (7, 8). Hip fractures in older adults require hospitalization and medical care, significantly burdening families, the healthcare system, and society financially (9–11). By 2050, hip fractures are expected to rise to between 7.3 and 21.3 million worldwide, with 5.9 million occurring in China (12). Consequently, the adoption of preventive measures and early intervention strategies is crucial for mitigating the impact of hip fracture incidence among the older population.

Frailty is increasingly recognized as a key indicator of older adults' health (13). Frailty is a complex syndrome characterized by diminished physiological reserves and an elevated risk of falls, fractures, disability, and hospitalization (14, 15). This condition represents the total health deficits accumulated over a person's life, typically measured by the Frailty Index (FI) (16, 17). A higher FI indicates more health deficiencies and greater frailty (18). Frailty challenges society and healthcare systems, not just affecting older people (19). Thus, it is a major public health challenge in aging societies (20). A study found that frailty, indicated by the FI, predicts future fractures in older adults, with frail individuals having a significantly higher risk of hip fractures than non-frail individuals (21). Conversely, a Brazilian study found no correlation between the FI and hip fracture, suggesting it may not be a reliable predictor of such outcomes (22). Therefore, the specific relationship between FI and hip fracture remains to be explored.

Anemia represents an additional clinical manifestation associated with aging, attributed to the decline in erythropoietin levels and the diminished hematopoietic reserves that occur with advancing age (23). In older adults, anemia is closely linked to physical dysfunction, reduced mobility, a higher risk of disability, hip fractures, and increased mortality (24–27). Taking these factors into account, anemia might be associated with hip fracture. Research in Korea found a link between anemia and increased fracture risk in older people. The study found that anemia was associated with a higher risk of hip fractures in both males and females, with the risk significantly elevated in those with moderate to severe anemia (28).

In this cross-sectional study, we hypothesized that the relationship between FI and hip fracture is mediated by anemia. Utilizing data from the China Health and Retirement Longitudinal Study (CHARLS). We investigated how anemia mediates the relationship between the FI and hip fracture among Chinese older adults, assessing the mediating role within key subgroups and considering the presence or absence of anemia.

## 2 Study methodology

### 2.1 Data collection

**Figure 1:**
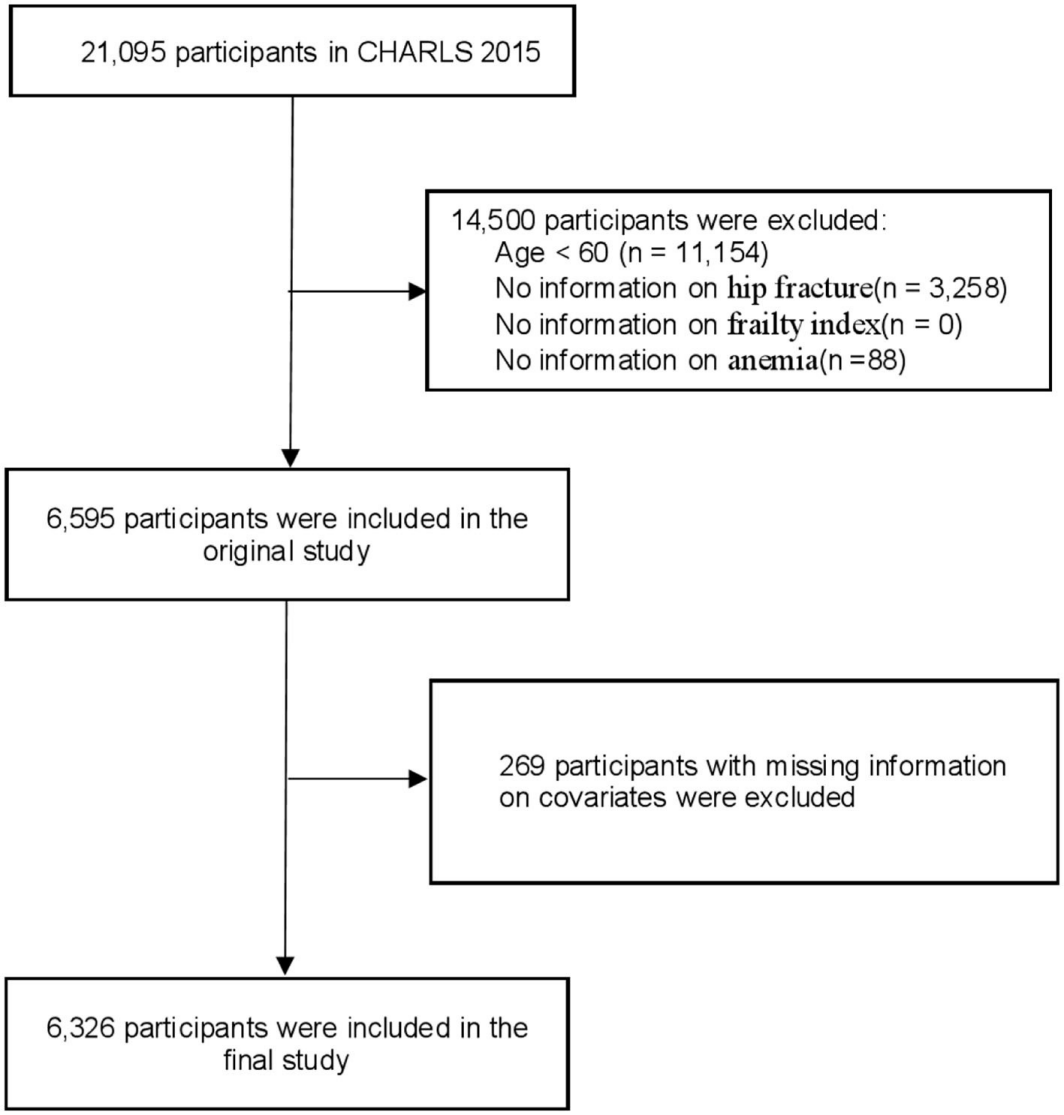
Flowchart of the participants selection process. CHARLS, China health and retirement longitudinal study.

This research employed a cross-sectional design using data from the CHARLS. CHARLS is a national longitudinal survey targeting Chinese residents aged 45 and older, designed to generate a high-quality, representative, and publicly accessible micro-database. This database provides comprehensive information on middle-aged and older individuals. Participants were chosen from 28 Chinese provinces using a multi-stage probability proportional to size (PPS) sampling technique. Detailed information regarding CHARLS has been documented in a previous study (29). The national baseline survey for CHARLS was conducted in 2015, encompassing 21,095 participants. Participants younger than 60 years (*n* = 11,154), those lacking hip fracture data (*n* = 3,258), FI data (*n* = 0), anemia data (*n* = 88), and those with missing covariates (*n* = 269) were excluded. As a result, a total of 6,326 older adults were eligible for analysis, with details provided in Figure 1.

### 2.2 Assessments

#### 2.2.1 Hip fracture

The primary outcome of interest in this study was the incidence of hip fractures. Hip fractures were evaluated based on self-reported answers to the query, “Have you fractured your hip since the last interview?” (30). To ensure accurate understanding, interviewers explained the anatomical location of the hip bone before the interview. Responses were classified as “Yes” or “No”, with the follow-up period ending in January 2016.

#### 2.2.2 Frailty index

In this study, the calculation of the FI follows a standardized methodology that incorporates all major health domains (32) items were chosen to construct the FI, encompassing variables such as comorbidities, physical function, disabilities, depression, and cognition (31). This FI has undergone validation using the CHARLS database and has been corroborated across various other cohorts (31–34). Except for the 32nd item, each item is converted into binary values of 0 or 1 based on specific cutoff criteria, where 0 indicates no deficit and 1 indicates a deficit. The 32nd item is a continuous variable between 0 and 1, where higher values indicate reduced cognitive abilities. Supplementary Table S1 offers additional information about the FI. For each participant, the 32-item FI (32-FI) is computed by dividing the sum of present health deficits by 32. As a result, FI values vary between 0 and 1, with higher scores indicating greater frailty. According to prior research, frailty is characterized by a 32-FI score of 0.25 or greater (35). The calculation of the 32-FI is performed for each wave of the CHARLS dataset.

To optimize data use and maintain indicator quality, we allowed up to 20% missing variables (a maximum of 6 items) in the FI calculation, a method frequently used in previous studies (19, 36). If participants have over six missing values for these indicators, their FI will be deemed missing. For participants with six or fewer missing values, the mean of the available scores will be computed. For instance, if a participant has 28 valid deficits with a cumulative score of 6, the FI would be computed as 6 divided by 28, resulting in an FI of 0.21.

#### 2.2.3 Anemia

Blood samples were obtained by qualified nursing personnel, and a complete blood count was conducted at the local health center. According to World Health Organization (WHO) standards, anemia is defined by hemoglobin levels below 12.0 g/dL for women and 13.0 g/dL for men (37).

#### 2.2.4 Control variables

The baseline survey included various covariates: gender, age, marital status (married or single), family residence (urban or rural), number of chronic conditions (≥2, 1, or 0), smoking status (smoker or non-smoker), alcohol consumption (drinker or non-drinker), and highest educational attainment (illiterate, primary or middle school, or high school and above).The prevalence of chronic diseases was evaluated by considering 14 self-reported non-communicable diseases (NCDs): dyslipidemia, cancer, hypertension, chronic lung disease, diabetes, liver disease, myocardial infarction, cerebrovascular accident, kidney disease, asthma, psychiatric disorders, gastrointestinal disorders, cognitive disorders, and arthritis. Anthropometric measurements, specifically height and weight, were collected using standardized protocols to compute the body mass index (BMI). Participants were classified into four BMI categories according to the WHO criteria: underweight (BMI <18.5 kg/m^2^), normal weight (BMI 18.5–24.9 kg/m^2^), overweight (BMI 25.0–29.9 kg/m^2^), and obese (BMI ≥30.0 kg/m^2^).

### 2.3 Statistical analysis

Total sample characteristics were reported using means, standard deviations and interquartile ranges for continuous variables, frequencies with percentages for categorical variables. Secondly, we performed Spearman's correlation analysis to assess the relationships among the primary variables. The study utilized the mediation model introduced by Baron and Kenny (38). This mediation model assessed the mediating role of baseline anemia in the relationship between baseline FI and hip fracture. Linear regression analyses were conducted to: (1) explore the association between FI and anemia, (2) examine the link between FI and hip fracture, and (3) investigate the FI-hip fracture relationship with anemia as a mediator. We utilized a nonparametric bootstrap approach with 1,000 resamples to assess both the total and indirect effects (39). Finally, using the adjustment variables in Model III, we used restricted cubic spline (RCS) fitting to investigate the potential nonlinear association between FI and hip fracture. In addition, gender, age, marital status, family residence, chronic conditions, smoking, alcohol consumption, and highest level of education and BMI were considered. Fourteen self-reported NCDS including dyslipidemia, cancer, hypertension, chronic pulmonary disease, diabetes, liver disease, myocardial infarction, cerebrovascular accident, kidney disease, asthma, psychiatric disease, gastrointestinal disease, cognitive impairment and arthritis were used to determine the prevalence of chronic conditions for subgroup analysis. These analyses aimed to determine whether these factors modulate the association between FI and anemia on hip fracture. A mediation effect is statistically significant if the bias-corrected and accelerated 95% confidence interval (CI)for each path coefficient does not include 0. R Version 4.3.2 was utilized for all analyses, with two-tailed p < 0.05 representing significance.

## 3 Result

### 3.1 Baseline characteristics of the study participants

Table 1 details the characteristics of study participants, categorized by the presence or absence of a hip fracture. At baseline, the sample consisted of 6,326 participants, of whom 6,167 had no hip fracture and 159 had hip fracture. Participants had a mean age of 68.0 years (SD = 6.5), with women comprising 50.3% of the group. Approximately 80.3% of participants achieved an educational level of Illiteracy. A significant proportion of older adults resided in urban areas (62.4%) and were married (79.6%). The hip fracture group had a significantly higher mean age compared to the non-hip fracture group (*P* < 0.001). A significant difference in marital status was observed between the two groups (*P* = 0.04), with a higher proportion of married individuals in the hip fracture group. Smokers comprised 47.3% of the overall population and 45.3% of those with hip fractures. Alcohol consumption was reported by 47.1% of the overall population and 49.7% of individuals with hip fractures. The mean FI for all participants was 0.2 (SD = 0.1). The mean FI for the hip fracture group was 0.3 (SD = 0.2). The hip fracture group exhibited a significantly higher FI compared to the other group (*P* < 0.001). Frailty was observed in 25.9% of the overall participants, while 49.7% of individuals within the hip fracture group exhibited frailty. A significant difference in frailty prevalence was observed between the groups (*P* < 0.001), with a higher incidence in the hip fracture group. Among the participants, 22.1% were diagnosed with anemia, in contrast to 36.5% within the hip fracture group. A significant difference in anemia prevalence was found between the groups (*P* < 0.001), with a higher rate in the hip fracture group. Moreover, the analysis of baseline characteristics of included and excluded participants is shown in Supplementary Table S2.

**Table 1:** Characteristics of the study participants by hip fracture or not.

**Variables**	**Total (*n* = 6,326)**	**Non-hip fracture (*n* = 6,167)**	**Hip fracture (*n* = 159)**	***P* value**
Age, mean ± SD	68.0 ± 6.5	67.9 ± 6.4	69.7 ± 6.6	< 0.001
Gender, *n* (%)				0.866
Male	3,145 (49.7)	3,067 (49.7)	78 (49.1)	
Female	3,181 (50.3)	3,100 (50.3)	81 (50.9)	
Marital status, *n* (%)				0.004
Married	5,037 (79.6)	4,925 (79.9)	112 (70.4)	
Unmarried	1,289 (20.4)	1,242 (20.1)	47 (29.6)	
Education levels, *n* (%)				0.159
Illiteracy	5,078 (80.3)	4,941 (80.1)	137 (86.2)	
Primary or middle school	862 (13.6)	846 (13.7)	16 (10.1)	
High school and above	386 (6.1)	380 (6.2)	6 (3.8)	
Residence, *n* (%)				0.263
Urban	3,949 (62.4)	3,843 (62.3)	106 (66.7)	
Rural	2,377 (37.6)	2,324 (37.7)	53 (33.3)	
Smoking, *n* (%)				0.606
No	3,334 (52.7)	3,247 (52.7)	87 (54.7)	
Yes	2,992 (47.3)	2,920 (47.3)	72 (45.3)	
Drinking, *n* (%)				0.502
No	3,349 (52.9)	3,269 (53)	80 (50.3)	
Yes	2,977 (47.1)	2,898 (47)	79 (49.7)	
BMI, *n* (%)				0.352
Underweight	496 (7.8)	483 (7.8)	13 (8.2)	
Normal	3,807 (60.2)	3,707 (60.1)	100 (62.9)	
Overweight	1,712 (27.1)	1,669 (27.1)	43 (27)	
Obesity	311 (4.9)	308 (5)	3 (1.9)	
Number of chronic conditions, *n* (%)				0.006
0	1,182 (18.7)	1,162 (18.8)	20 (12.6)	
1	1,428 (22.6)	1,402 (22.7)	26 (16.4)	
≥2	3,716 (58.7)	3,603 (58.4)	113 (71.1)	
Frailty index, mean ± SD	0.2 ± 0.1	0.2 ± 0.1	0.3 ± 0.2	< 0.001
Frailty, *n* (%)				< 0.001
No	4,689 (74.1)	4,609 (74.7)	80 (50.3)	
Yes	1,637 (25.9)	1,558 (25.3)	79 (49.7)	
Anemia, *n* (%)				< 0.001
No	4,925 (77.9)	4,824 (78.2)	101 (63.5)	
Yes	1,401 (22.1)	1,343 (21.8)	58 (36.5)	

### 3.2 The relationship between important variables

Table 2 presents the relationship between FI, anemia, and hip fracture. The study evaluated the relationship between FI and hip fracture in 6,326 participants. In the unadjusted model, the odds ratio (OR) for the association between FI and hip fracture was 1.13 (95% CI: 1.10–1.16, *p* < 0.001), demonstrating a significant positive relationship. In models 1, 2, and 3, the OR was 1.13. 95% CI: 1.09–1.16 (model 1); 1.10–1.16 (model 2); 1.09–1.16 (model 3). All *p* < 0.001. This indicated that adjustment for related factors did not significantly alter the significant positive relationship between FI and hip fracture.

**Table 2:** Logistic regression results of frailty index and anemia with hip fracture.

**Variables**	**No**	**Unadjusted**		**Model 1**		**Model 2**		**Model 3**	
		**OR (95% CI)**	***p*** **value**	**OR (95% CI)**	***p*** **value**	**OR (95% CI)**	***p*** **value**	**OR (95% CI)**	***p*** **value**
Frailty index	6,326	1.13 (1.10–1.16)	< 0.001	1.13 (1.09–1.16)	< 0.001	1.13 (1.10–1.16)	< 0.001	1.13 (1.09–1.16)	< 0.001
**Frailty**									
No	4,689	1 (Ref)		1 (Ref)		1 (Ref)		1 (Ref)	
Yes	1,637	2.92 (2.13–4.01)	< 0.001	2.80 (2.02–3.88)	< 0.001	2.90 (2.09–4.03)	< 0.001	2.65 (1.87–3.76)	< 0.001
**Anemia**									
No	4,925	1 (Ref)		1 (Ref)		1 (Ref)		1 (Ref)	
Yes	1,401	2.06 (1.49–2.86)	< 0.001	1.8 (1.34–2.62)	< 0.001	1.88 (1.33–2.64)	< 0.001	1.88 (1.33–2.64)	< 0.001

Model 1: adjusted for age, gender, educational level, marital status, and residence. Model 2: adjusted for model 1 + smoking status, drinking status, and BMI. Model 3: adjusted for model 2 + 14 chronic diseases. OR, odds ratio; 95% CI, 95% confidence interval. OR, odds ratio; CI, confidence interval.

The study comprised a cohort of 1,637 individuals who had been diagnosed with frailty. In the unadjusted model, frailty was significantly associated with hip fracture, with an odds ratio of 2.92 (95% CI: 2.13–4.01, *p* < 0.001). In models 1, 2 and 3, the OR were 2.80, 2.90 and 2.65, respectively. 95% CI: 2.02–3.88 (model 1); 2.09–4.03 (model 2); 1.87–3.76 (model 3), all *p* < 0.001. The positive correlation between frailty and hip fracture persisted significantly even after adjusting for relevant factors.

The study included 1,401 participants diagnosed with anemia. In the unadjusted model, the link between anemia and hip fracture among participants was OR = 2.06, 95% CI: 1.49–2.86, *p* < 0.001, suggesting a significant positive association. In models 1, 2 and 3, the OR were 1.80, 1.88 and 1.88, respectively. 95% CI: 1.34–2.62 (model 1); 1.33–2.64 (model 2); 1.33–2.64 (model 3), all *p* < 0.001. The positive correlation between anemia and hip fracture persisted significantly even after adjusting for relevant factors.

**Figure 2:**
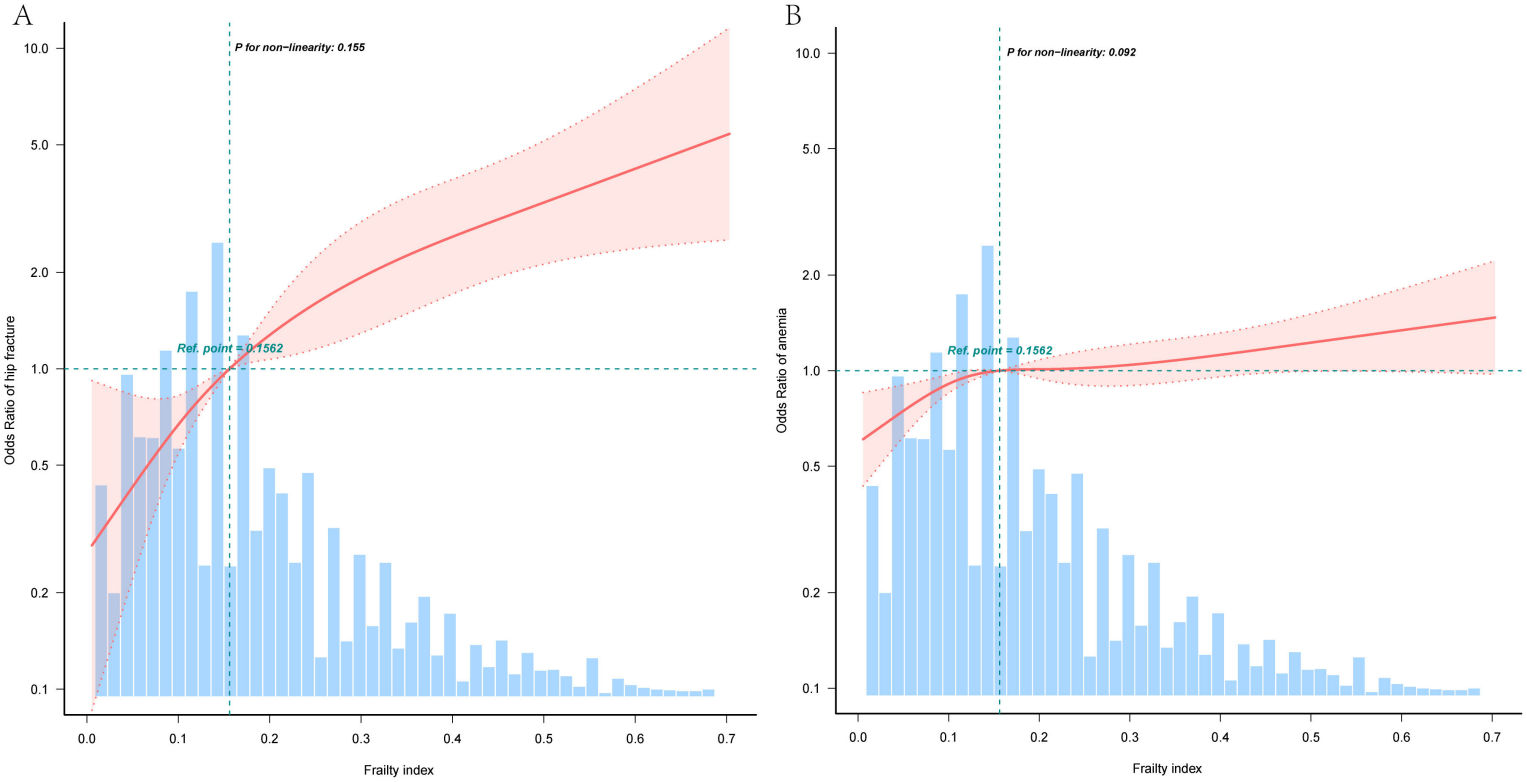
Association between frailty index and hip fracture **(A)**; Association between frailty index and anemia **(B)**. Solid and dashed lines represent the predicted value and 95% CI. Blue bars represent the distribution of the entire cohort. Adjustments were made for age, gender, educational level, marital status, residence, smoking status, drinking status, BMI, and 14 chronic diseases, only 95% of the data is displayed. BMI, Body Mass Index.

RCS regression analysis showed that FI was linearly related to hip fracture (for nonlinearity, *P* = 0.155; Figure 2A). Meanwhile, RCS regression analysis showed that FI was also linearly related to anemia (for nonlinearity, *P* = 0.092; Figure 2B).

### 3.3 Interactive effects of frailty index and anemia on hip fracture

Table 3 presents the combined effect of frailty and anemia on hip fracture after adjusting for confounding factors. Individuals with both frailty and anemia have a significantly higher risk of hip fracture (OR was 4.61, 95%CI 2.80–7.61) compared to those without these conditions. However, there was no multiplicative and additive interactions between frailty and anemia on hip fracture (Additive: relative excess risk of interaction was 0.81, 95% CI: −1.28–2.89; attributable proportion due to Interaction was 0.17, 95% CI: −0.22–0.57; Multiplicative, OR was 0.82, 95% CI 0.42–1.59).

**Table 3:** Interactive effects of frailty index and anemia on hip fracture.

**Frailty status**	**Anemia status**	**OR (95% CI)**	**Multiplicative scale**	**Measures of additive interaction**	
			**OR (95% CI)**	**RERI (95%CI)**	**AP (95%CI)**
Non-frailty	Non-anemia	1	0.82(0.42–1.59)	0.81(−1.28–2.89)	0.17(−0.22–0.57)
Non-frailty	Anemia	2.01 (1.25–3.22) ^**^			
Frailty	Non-anemia	2.80 (1.83–4.28) ^**^			
Frailty	Anemia	4.61 (2.80–7.61) ^**^			

Adjusted Model: adjust for all of these variables, including age, gender, educational level, marital status, residence, smoking status, BMI, and 14 chronic diseases. OR, odds ratio; 95% CI, 95% confidence interval; RERI, relative excess risk of interaction; AP, attributable proportion due to interaction. ^**^P < 0.01.

### 3.4 Anemia mediates the association between frailty index and hip fracture

Table 4 illustrates the association between FI, anemia, and hip fractures. The findings indicate a positive association between FI and hip fracture (r = 0.11, *P* < 0.001). Additionally, FI exhibits a positive association with anemia (r = 0.06, *P* < 0.001), and anemia exhibits a positive association with hip fracture (r = 0.06, *P* < 0.001).

**Table 4:** Association among frailty index and anemia with hip fracture.

**Variables**	**Frailty index**	**Anemia**	**Hip fracture**
Frailty index	1.00		
Anemia	0.06^***^	1.00	
Hip fracture	0.11^***^	0.06^***^	1.00

Adjusted Model: adjust for all of these variables, including age, gender, educational level, marital status, residence, smoking status, BMI, and 14 chronic diseases. ^***^P-value < 0.001.

**Figure 3:**
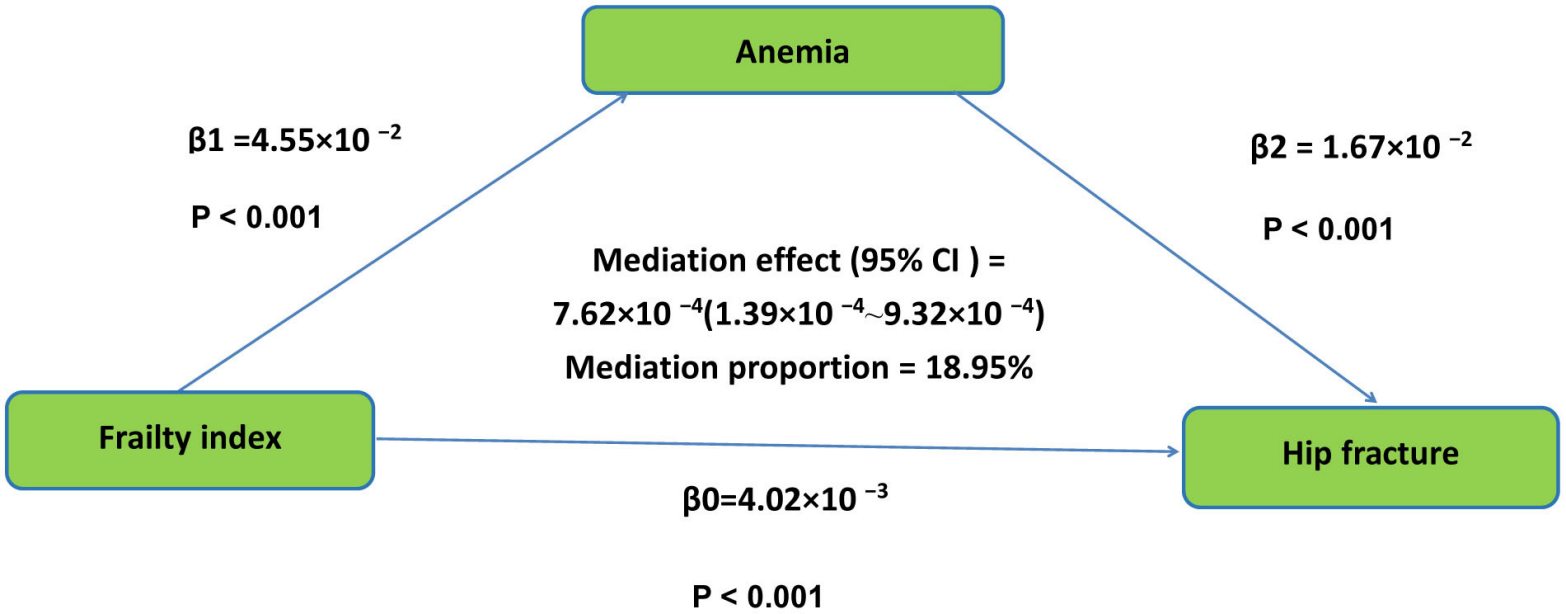
Mediation models of frailty index, anemia, and hip fracture in older Chinese adults. β0 was the total effect of frailty index on hip fracture; β1 represents the effect of frailty index on anemia; β2 represents the effect of anemia on hip fracture. The mediation effect was computed as the product of “β1” and “β2”(β1 × β2), and the mediation proportion was calculated as the ratio of the mediation effect product to total effects [(β1 × β2)/β0].

Utilizing bootstrap analysis, the influence of FI on hip fracture was determined to be 4.02 × 10^−3^ at baseline, *P* < 0.001. The 95% CI ranged from 1.39 × 10^−4^ to 9.32 × 10^−4^. The findings indicate that anemia substantially mediated the association between FI and hip fracture, explaining 18.95% of the total effect variance. The mediation pathway is depicted in Figure 3.

## 4 Discussion

This cross-sectional study is the first to examine how anemia mediates the relationship between FI and hip fracture in a population-based cohort of older Chinese individuals. The results demonstrate a significant association between baseline FI and hip fracture. Anemia partially mediated the link between FI and hip fracture, confirming our initial hypothesis.

FI is associated with bone health, as studies show frail individuals often have lower bone mineral density, increasing their fracture risk (40). Previous study indicated that older adults diagnosed as frail using a frailty assessment tool faced a significantly increased risk of hip fractures compared to robust individuals, highlighting frailty as a predictor of fracture risk (41). Meanwhile, a study on older adults found that higher FI scores increased hip fracture risk, highlighting frailty's importance in assessing fracture risk (42). A study of HIV-infected male veterans identified the FI as a significant predictor of fragility fractures, including hip fractures, demonstrating its wide clinical relevance (43). Overall, the FI serves as a valuable indicator for identifying individuals at elevated risk of hip fracture and for informing targeted risk reduction strategies. Its link to hip fractures highlights the importance of regular frailty assessments in older adults to improve fracture prevention and management (40, 42–45).

A study indicates that frailty may predict anemia in older adults, as it is linked to weakness, exhaustion, and low physical activity, which can lower hemoglobin levels and raise anemia risk (46). In addition, previous studies indicate that frailty, linked to age-related declines in body reserves, is associated with various diseases, including anemia (45). A prior investigation into the interrelationship among erythrocyte indices, anemia, and obesity-related diseases identified a significant association between frailty and anemia within the context of metabolic disorders. This underscores the multifactorial nature of frailty and emphasizes the necessity of considering anemia as a contributing factor to various health conditions (47). Malnutrition, marked by insufficient iron, vitamin B12, and folic acid intake, commonly links anemia and frailty. Malnutrition can lead to muscle wasting and weakness, which are key traits of frailty. It can also hinder red blood cell production, resulting in anemia (48, 49). Another study examined the link between FI and blood-derived inflammatory markers, finding that elevated systemic inflammation, common in anemia, significantly correlates with increased FI. This indicates that inflammation might connect frailty and anemia (50). A study indicates that inflammatory markers like C-reactive protein (CRP) and interleukin-6 (IL-6) are elevated in frail individuals and linked to anemia and poor health outcomes. It suggests inflammation may worsen frailty by causing muscle wasting and reduced physical function, which are common in older adults with anemia (51).

A Korean study found that anemia increases fracture risk, especially hip fractures, in older adults. The risk is notably higher in those with moderate-to-severe anemia, highlighting the impact of anemia severity on fracture risk (28). Research from the Women's Health Initiative indicates that postmenopausal women with anemia have a significantly increased risk of hip fractures compared to those without anemia, emphasizing the importance of recognizing anemia as a fracture risk factor in older women (52). A study on cardiovascular health identified a correlation between anemia and an increased risk of hip fracture in males, with a similar association observed between declining hemoglobin levels and hip fracture risk (53). A Taiwanese study found that iron deficiency anemia (IDA) significantly increases the risk of osteoporosis, which subsequently raises the likelihood of fractures, including hip fractures (54).

Anemia was found to mediate the relationship between FI and hip fractures, supporting the idea that FI may promote hip fractures. A study indicated that low hemoglobin and anemia independently raise frailty risk, implying anemia could both result from and contribute to frailty (55). Furthermore, the correlation between systemic inflammation and anemia indicates a positive association between inflammatory markers and anemia. Inflammatory cytokines like IL-6 contribute to anemia by promoting hepcidin production, which restricts intestinal iron absorption and sequesters iron in macrophages, resulting in IDA (56). This underscores the possibility that inflammatory processes linked to frailty may exacerbate or contribute to the onset of anemia (57). Evidence indicates that anemia significantly impacts hip fracture incidence and outcomes (58). Anemia influences certain inflammatory mediators, such as CRP, which, when elevated, are linked to higher mortality in hip fracture patients, indicating that anemia-related inflammation may lead to negative outcomes (59). Research shows that red cell distribution width (RDW), an indicator of red blood cell size variation, can predict hip fracture risk in older men without anemia, emphasizing the influence of minor red blood cell variations on bone health and fracture susceptibility (27).

A holistic approach combining nutritional, physical, and medical interventions is essential to prevent frailty, anemia, and hip fractures in older people. Essential calcium and vitamin D supplements significantly reduce hip fracture risk, especially in older and institutionalized people (60). Exercise is another key component in preventing frailty and fractures. Engaging in regular physical activity, particularly resistance and weight-bearing exercises, enhances muscle strength, balance, and bone density, which reduces the risk of falls and fractures (61). Anemia in older people can be effectively addressed through dietary modifications, supplementation, and, when necessary, medical interventions such as erythropoiesis-stimulating agents or iron therapy (62–64).

This research has certain limitations. Firstly, the hip fracture data were obtained from patients' self-reported responses, which may be influenced by recall bias. Secondly, the characteristics of included and excluded participants were found to have significant differences among some covariables, including age, marital status, education level, place of residence, BMI, and the number of chronic diseases. This exclusion process may introduce selection bias. Thirdly, while this study accounted for several covariates, there remains the possibility that potential confounding factors, such as nutritional status and acute infection, could still be present and may influence the conclusions. Fourthly, the study did not examine the impact of anemia severity and type on the relationship between frailty and hip fracture, which may compromise the precision of the conclusions drawn. Fifthly, the cross-sectional nature of this study limits the ability to establish causal links between the FI, anemia, and hip fracture. Finally, our study was limited to 2015 data; future research should investigate additional pathways and employ repeated measures over an extended period.

## 5 Conclusion

This cross-sectional study analyzed 2015 CHARLS data to evaluate the overall impact of the FI on hip fractures and the mediating role of anemia. The result showed that FI and anemia were positively associated with hip fracture, and anemia played a mediating role in the association between FI and hip fracture. Reducing the FI and preventing anemia in the older people are crucial strategies for mitigating the risk of hip fractures. To promote healthy aging and prevent hip fractures, it is essential to encourage regular nutritional supplementation, engage in both aerobic and resistance training, and implement effective medical management of anemia among older adults.

## Data Availability

Publicly available datasets were analyzed in this study. This data can be found here: https://charls.pku.edu.cn/.
