# Supplementary material for: Anemia as a mediator: bridging the frailty index and hip fractures in older Chinese populations
Source: Front Public Health. 2025 Apr 23;13:1558074. doi: 10.3389/fpubh.2025.1558074 (PMC12055539; doi:10.3389/fpubh.2025.1558074)
Supplement: Supplementary file 1 [file Data_Sheet_1.zip › Supplementary Tables/Table S1.docx]

**Table S1.** **Variables Used to Construct the Frailty Index.**

| **No** | **Description of the items** | **Cut-off value** |
| --- | --- | --- |
| 1 | Self-reported diagnosis of hypertension by a doctor | Yes = 1, No = 0 |
| 2 | Self-reported diagnosis of diabetes by a doctor |  |
| 3 | Self-reported diagnosis of heart attack, coronary heart disease, angina, congestive heart failure, or other heart problems by a doctor |  |
| 4 | Self-reported diagnosis of stroke by a doctor |  |
| 5 | Self-reported diagnosis of cancer by a doctor |  |
| 6 | Self-reported diagnosis of arthritis by a doctor |  |
| 7 | Self-reported diagnosis of chronic lung diseases by a doctor |  |
| 8 | Self-reported diagnosis of asthma by a doctor |  |
| 9 | Self-reported diagnosis of emotional, nervous, or psychiatric problems by a doctor |  |
| 10 | Self-reported diagnosis of memory-related disease by a doctor |  |
| 11 | Self-reported vision problems |  |
| 12 | Self-reported hearing problems |  |
| 13 | Difficulty with dressing | Did not have any problems with the activity=0; some difficulty with the activity or could not do the activity=1. |
| 14 | Difficulty with bathing or showering |  |
| 15 | Difficulty with eating |  |
| 16 | Difficulty with getting in and out of bed |  |
| 17 | Difficulty with using the toilet |  |
| 18 | Difficulty with managing money |  |
| 19 | Difficulty with taking medications |  |
| 20 | Difficulty with shopping for groceries |  |
| 21 | Difficulty with preparing meals |  |
| 22 | Difficulty with doing housework |  |
| 23 | Difficulty with walking 100 yards |  |
| 24 | Difficulty with getting up from a chair after sitting for long periods |  |
| 25 | Difficulty with climbing several flights of stairs without resting |  |
| 26 | Difficulty with lifting or carrying weights over 10 pounds/ jins |  |
| 27 | Difficulty with picking up a coin from the table |  |
| 28 | Difficulty with stooping, kneeling, or crouching |  |
| 29 | Difficulty with reaching arms above shoulder level |  |
| 30 | Self-reported health | Poor/ fair = 1, excellent/ very good/ or good = 0 |
| 31 | Depressive symptoms: CESD-10 questionnaire | CESD-10 >10 =1, ≤10 =0 |
| 32 | Cognition: (memory test score + orientation test score) **/** 14 | Continuous, ranging from 0 to 1 |

Abbreviations: CES-D, Center for Epidemiologic Studies Depression Scale
