# Supplementary material for: Anemia as a mediator: bridging the frailty index and hip fractures in older Chinese populations
Source: Front Public Health. 2025 Apr 23;13:1558074. doi: 10.3389/fpubh.2025.1558074 (PMC12055539; doi:10.3389/fpubh.2025.1558074)
Supplement: Supplementary file 1 [file Data_Sheet_1.zip › Supplementary Tables/Table S2.doc]

Table S2. Characteristics of the study participants

| Variables | Total  (n = 9941) | Exclude participants  (n = 3615) | Included participants  (n = 6326) | p |
| --- | --- | --- | --- | --- |
| Age, Mean ± SD | 68.5 ± 7.1 | 69.5 ± 7.9 | 68.0 ± 6.5 | < 0.001 |
| Gender, n (%) |  |  |  | 0.245 |
| Male | 4896 (49.3) | 1751 (48.5) | 3145 (49.7) |  |
| Female | 5040 (50.7) | 1859 (51.5) | 3181 (50.3) |  |
| Marital status, n (%) |  |  |  | < 0.001 |
| Married | 7772 (78.3) | 2735 (76) | 5037 (79.6) |  |
| Unmarried | 2152 (21.7) | 863 (24) | 1289 (20.4) |  |
| Education levels, n (%) |  |  |  | < 0.001 |
| Illiteracy | 7877 (79.3) | 2799 (77.7) | 5078 (80.3) |  |
| Primary or middle school | 1321 (13.3) | 459 (12.7) | 862 (13.6) |  |
| High school and above | 732 ( 7.4) | 346 (9.6) | 386 (6.1) |  |
| Residence, n (%) |  |  |  | < 0.001 |
| Urban | 5978 (60.1) | 2029 (56.1) | 3949 (62.4) |  |
| Rural | 3963 (39.9) | 1586 (43.9) | 2377 (37.6) |  |
| Smoking, n (%) |  |  |  | 0.193 |
| Yes | 5248 (53.2) | 1914 (54.1) | 3334 (52.7) |  |
| No | 4618 (46.8) | 1626 (45.9) | 2992 (47.3) |  |
| Drinking, n (%) |  |  |  | 0.168 |
| Yes | 5265 (53.5) | 1916 (54.4) | 3349 (52.9) |  |
| No | 4584 (46.5) | 1607 (45.6) | 2977 (47.1) |  |
| BMI, n (%) |  |  |  | < 0.001 |
| Underweight | 660 ( 8.4) | 164 (10.7) | 496 (7.8) |  |
| Normal | 4769 (60.7) | 962 (62.6) | 3807 (60.2) |  |
| Overweight | 2061 (26.2) | 349 (22.7) | 1712 (27.1) |  |
| Obesity | 372 ( 4.7) | 61 (4) | 311 (4.9) |  |
| Number of chronic conditions, n (%) |  |  |  | < 0.001 |
| 0 | 2013 (20.2) | 831 (23) | 1182 (18.7) |  |
| 1 | 2227 (22.4) | 799 (22.1) | 1428 (22.6) |  |
| ≥2 | 5701 (57.3) | 1985 (54.9) | 3716 (58.7) |  |
